# Strain-Specific Identification and In Vivo Immunomodulatory Activity of Heat-Killed *Latilactobacillus sakei* K040706

**DOI:** 10.3390/foods10123034

**Published:** 2021-12-07

**Authors:** Kyung-Sook Chung, Jae Woong Choi, Ji-Sun Shin, Seo-Yeon Kim, Hee-Soo Han, Su-Yeon Kim, Kwang-Young Lee, Joo-Yeon Kang, Chang-Won Cho, Hee-Do Hong, Young Kyoung Rhee, Kyung-Tae Lee

**Affiliations:** 1 Department of Pharmaceutical Biochemistry, College of Pharmacy, Kyung Hee University, Seoul 02447, Korea; adella76@khu.ac.kr (K.-S.C.); jsunvet@naver.com (J.-S.S.); tjdus7772@hanmail.net (S.-Y.K.); heesu3620@daum.net (H.-S.H.); dlstm4@gmail.com (S.-Y.K.); gloryi@naver.com (K.-Y.L.); 2 Korea Food Research Institute, 245, Nongsaengmyeong-ro, Iseo-myeon, Wanju-gun 55365, Jeollabuk-do, Korea; choijw@kfri.re.kr (J.W.C.); cwcho@kfri.re.kr (C.-W.C.); honghd@kfri.re.kr (H.-D.H.); 3 Department of Life and Nanopharmaceutical Sciences, Graduate School, Kyung Hee University, Seoul 02447, Korea; 4 NOVAREX Co., Ltd., 94, Gangni 1-gil, Ochang-eup, Cheongwon-gu, Cheongju-si 363-885, Chungcheongbuk-do, Korea; kjy@novarex.co.kr

**Keywords:** heat-killed *Latilactobacillus sakei*, immune improvement, cyclophosphamide, microbiota, splenocyte

## Abstract

We previously reported that the immunostimulatory activity of heat-killed *Latilactobacillus sakei* K040706 in macrophages and cyclophosphamide (CTX)-treated mice. However, identification of heat-killed *L. sakei* K040706 (heat-killed LS06) using a validated method is not yet reported. Further, the underlying molecular mechanisms for its immunostimulatory effects in CTX-induced immunosuppressed mice remain unknown. In this study, we developed strain-specific genetic markers to detect heat-killed *L. sakei* LS06. The lower detection limit of the validated primer set was 2.1 × 10^5^ colony forming units (CFU)/mL for the heat-killed LS06 assay. Moreover, oral administration of heat-killed LS06 (10^8^ or 10^9^ CFU/day, p.o.) effectively improved the body loss, thymus index, natural killer cell activity, granzyme B production, and T and B cell proliferation in CTX-treated mice. In addition, heat-killed LS06 enhanced CTX-reduced immune-related cytokine (interferon-γ, interleukin (IL)-2, and IL-12) production and mRNA expression. Heat-killed LS06 also recovered CTX-altered microbiota composition, including the phylum levels of Bacteroidetes, Firmicutes, and Proteobacteria and the family levels of *Muribaculaceae*, *Prevotellaceae*, *Tannerellaceae*, *Christensenellaceae*, *Gracilibacteraceae*, and *Hungateiclostridiaceae*. In conclusion, since heat-killed *L. sakei* K040706 ameliorated CTX-induced immunosuppression and modulated gut microbiota composition, they have the potential to be used in functional foods for immune regulation.

## 1. Introduction

Immunomodulators contribute to our immunity by activating immune cells [1], and can also act as immune stimulators to protect against extracellular parasites, bacteria, allergens, and toxins [2,3], or to reduce the side effects of drug-induced immunosuppression [4]. T helper (Th) cells play a pivotal role in immunomodulation [5]. Type 1 interferon (IFN)-γ-producing Th (Th1) cells help in mounting a host defense against intracellular pathogens, including protozoa, bacteria, and viruses, and are involved in the development of certain types of autoimmune diseases [2]. In contrast, type 2 interleukin (IL)-4/IL-5/IL-13-secreting Th (Th2) cells defend against helminth infections and venom exposure and participate in different types of allergic diseases, including asthma, atopic dermatitis, allergic rhinitis, and food allergy. Increasing evidence indicates that natural immune stimulators such as probiotic strains could be an alternative to conventional therapies for maintaining immune homeostasis [6].

Lactic acid bacteria (LAB), members of the order *Lactobacillales*, are probiotic bacteria that distinctively produce lactic acid. LAB are commonly found in certain foods (such as yogurt) and human and animal digestive and genital systems. The specific strains of LAB can be detected using the 16S rRNA gene sequencing [7,8,9]. However, a limitation of this method is the low resolution between closely-related species. Therefore, better strain-specific detection of LAB is critical for its precise identification and quantification. In the previous study, we developed a species-specific primer set for the identification of *Latilactobacillus sakei* [10]. This primer set had specificity and sensitivity at a species level. For the protection and monitoring of strain, we needed a primer set at a strain level.

We previously reported the presence of *Latilactobacillus sakei* K040706 (formerly known as *Lactobacillus sakei* K040706) in the Doenjang, which is made from Meju (a brick of fermented soybeans) in brine. Heat-killed *L. sakei* K040706 (heat-killed LS06) enhances the phagocytic ability of recombinant IFN-γ(rIFN-γ)-primed macrophages by upregulating the production of nitric oxide, tumor necrosis factor (TNF)-α, and IL-6 via activating the nuclear factor kappa-light-chain-enhancer of activated B cells and Toll-like receptor 2. It also restores immunological parameters in cyclophosphamide (CTX)-induced immunosuppressed mice [11]. In addition, live LS06 exerts an improvement effect of colitis, involving inflammatory mediator suppressions and microbiota composition [12], and stimulates the cytokine, hematopoiesis, and immune cell production in CTX-treated mice [13]. Although we have previously demonstrated the stimulating activity of heat-killed LS06 in immune organs indexes of CTX-treated mice, the role of immunomodulation involving the molecular mechanism of heat-killed LS06 is not fully elucidated. Therefore, in this study, we developed a strain-specific real-time polymerase chain reaction (RT-PCR) method for identifying heat-killed LS06 and then examined its immunostimulatory activity in CTX-treated mice.

## 2. Materials and Methods

### 2.1. Preparation of Heat-Killed LS06 and Genomic DNA

The bacterial strains, including *L. sakei* K040706 (KCCM11472P) and other lactic acid bacteria, are used in this study (Table 1). *Latilactobacillus* strains were inoculated in fresh MRS media (De Man, Rogosa and Sharpe agar, BD Bioscience, Franklin Lakes, NJ, USA) at 30 °C for 20 h in an aerobic condition. The cultured fraction of cells (1/100) was transferred into fresh MRS media. After overnight cultivation, 1 mL of the culture was applied for DNA isolation and the pellet was collected by centrifugation at 13,000× *g* for 15 min, 4 °C. After washing with PBS (pH 7.0) twice, heat inactivation was carried out at 121 °C for 15 min and freeze drying was performed. DNA extraction was performed with DNeasy Blood & Tissue kits (Qiagen, Hilden, Germany) according to the manufacturer’s instruction with the following modifications: the cultivated cells were lysed with lysozyme from chicken egg white (Sigma-Aldrich, Burlington, MA, USA) for 2 h at 37 °C.

### 2.2. Whole Genome Sequencing and Comparative Genomic Analysis

Genomic DNA of *L. sakei* K040706 was sequenced by single-molecule, real-time sequencing technology (SMRT) on a PacBio RS II instrument (Macrogen Inc., Daejeon, Republic of Korea). Genome assembly was achieved using Hierarchical Genome Assembly Process (HGAP) version 3.0 from the SMRT portal version 2.3, with default parameters, by Macrogen, Inc. The assembled sequence was submitted, and pan-genome orthologous groups (POGs) were constructed using the EzBioCloud Comparative Genomics Database (http://cg.ezbiocloud.net, accessed on 12 March 2021). Average nucleotide identity (ANI) values and Venn diagram also were calculated and constructed by the EzBioCloud database, respectively [14]. The whole genome sequence of *L. sakei* K040706 was listed on NCBI database (https://www.ncbi.nlm.nih.gov/sra/SRR15647685, accessed on 26 August 2021).

### 2.3. Design of Strain-Specific Primer

Primers are synthesized by Macrogen (Daejeon, Republic of Korea). The strain-specific marker bands were found by analysis of POGs. After selecting the closest 4 strains by the OrthoANI algorithm (Table 2), a complementary set of *L. sakei* K040706 was isolated. Three primer sequences sets were designed using the sequences in these regions. The primer pairs have a similar melting temperature (T_m_) and PCR product sizes (100 ~ 150 bp), which show a similar resolution by agarose gel electrophoresis. Primer-BLAST (http://www.ncbi.nlm.nih.gov/tools/primer-blast/, accessed on 26 August 2021) was applied to design strain-specific primer.

### 2.4. Design of Animal Experiments

Male ICR mice (*n* = 50, 6 weeks old) from Central Lab. Animal Inc. (Seoul, Republic of Korea) were kept at a temperature of 20 ± 5 °C, the humidity of 40–60%, and 12 h light/dark cycle environment before use. Animal experiments were approved by the animal care committee of Kyung Hee University (KHUASP(SE)-18-158).

**Figure 1:**
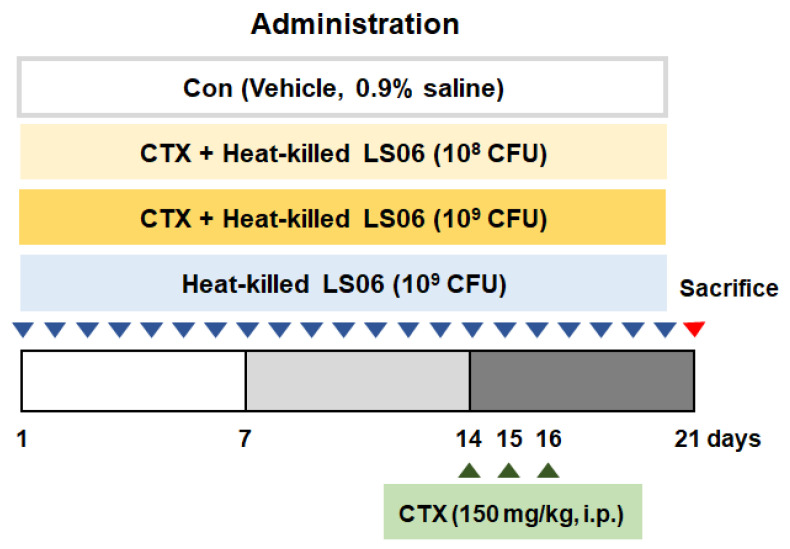
Animal study design. Establishment of cyclophosphamide (CTX)-induced immunosuppressed model and oral administration of heat-killed *L. sakei* K040706 (heat-killed LS06) 10^8^ or 10^9^ colony forming units (CFU)/day) for 20 days.

The CTX-induced immunosuppressed model was performed with some modifications [15,16]. Mice receive CTX (150 mg/kg/day, intraperitoneal (i.p.)) for immunosuppression on days 14, 15, and 16. After being acclimated for a week, mice were randomly allocated as follows (*n* = 10): (i) Con group (vehicle-treated control group, 0.9% saline), (ii) CTX group (vehicle + CTX 150 mg/kg), (iii) CTX + heat-killed LS06 10^8^ colony forming units (CFU) group, (iv) CTX + heat-killed LS06 10^9^ CFU group, (v) heat-killed LS6 10^8^ CFU group. Vehicle and heat-killed LS06 (10^8^ CFU or 10^9^ CFU/day) were orally supplemented for 20 days (Figure 1).

### 2.5. Preparation of Splenocytes and Assessment of NK Cytotoxic Activity

After sacrifice at the end of experiments, splenocytes were isolated and NK cell was activated [15]. NK cytotoxic activity was determined by measuring lactate dehydrogenase (LDH) levels, as performed in a previous report [13].

### 2.6. Determination of T Cell and B Cell Proliferation

To activate T cell or B cell, splenocytes were treated with concanavalin A (Con A, 5 μg/mL) or lipopolysaccharide (LPS, 1 μg/mL), respectively. The cell proliferation assay was assessed by using the Cell Titer 96^®^ Aqueous One Solution Reagent according to the manufacturer’s instructions (Promega, Madison, WI, USA).

### 2.7. Determination of Cytokine and Granzyme B Production

After T and B cell activation, the productions of cytokine and granzyme B in cell culture supernatants were quantified using ELISA kits (R&D systems, Minneapolis, MN, USA).

### 2.8. Determination of mRNA Expression

Splenic RNA was extracted by using Trizol reagent (Thermo Fisher Scientific, Waltham, MA, USA), and the level of Th1-related cytokines and *β-actin* mRNA expression was determined using quantitative RT-PCR (qRT-PCR) [11]. The oligonucleotide primers are listed in Supplemental Table S1.

### 2.9. Microbiota Profiling

Total genomic DNA from feces was prepared by using an *i*-genomic stool plus kit (Intron Biotechnology, Seoul, Korea). After the preparation and amplification of the library, selection of 16S rRNAs and taxonomic assignment were performed as in a previous report [11].

### 2.10. Statistical Analysis

Statistical analysis was performed by GraphPad Prism 5.01 software (La Jolla, CA, USA). Data are expressed as the mean ± standard error of the mean (SEM) (*n* = 10). Statistical differences were analyzed using one-way analysis of variance (ANOVA) and the Dunnett’s post hoc test, and *p* < 0.05 was indicated statistically significant.

## 3. Results

### 3.1. Screening of Strain-Specific Genetic Markers and Primer Design

**Figure 2:**
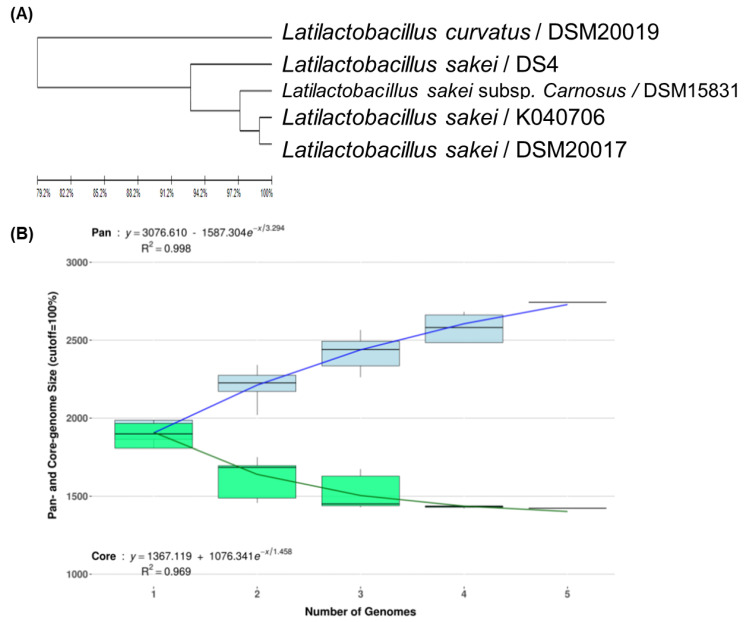

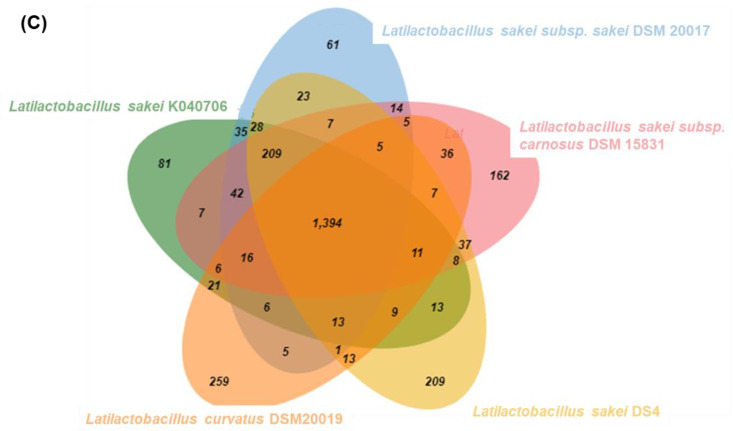
(**A**) Phylogenetic tree showing the relatedness of *L. sakei* strains based on ANI analysis. (**B**) Core and pan-genome curves show the trend of the core gene and pan-gene families with an increase in the number of genomes by Heap’s law model. (**C**) Pan-genome Venn diagram of *L. sakei* strains, including K040706, DSM 20017, DSM 15831, DS4, and DSM20019, showing the distribution of shared and unique pangenome orthologous groups (POGs) among the five strains.

To develop strain-specific genetic markers for LAB strains, we performed a comparative genomic analysis of *L. sakei* K040706 with four other closely related *Latilactobacillus* strains. The complete genome of *L. sakei* K040706 contained 1,977,298 sequences. A phylogenetic tree was constructed using an up-to-date bacterial core gene set (UBGC) to compare 92 core genes [17]. Three strains belonging to *L. sakei* and one belonging to *Latilactobacillus curvatus* were selected for the pan-genome analysis (Figure 2A). For the pan-genome analysis, Heap’s law regression model was applied, which showed a robust fit model with a clear upward trend and R^2^ = 0.998 (Figure 2B). The core genomes had 1,394 coding sequences (CDS), from which only 81 of the *L. sakei* K040706 were unique. These CDSs were not part of the plasmids or phage sequences. After comparative genomics, the unique CDSs were searched in the NCBI database to compare with the CDSs of other bacteria, including *Latilactobacillus* sp. Subsequently, three sequences were selected, and each primer set was checked using Primer-BLAST in NCBI (Table 2).

### 3.2. Specificity and Efficiency of Strain-Specific Real-Time PCR Assay

**Figure 3:**
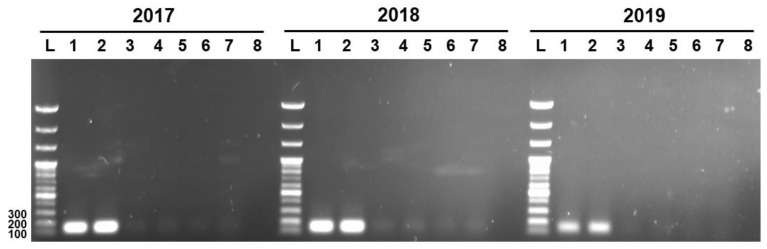
Detection of PCR product by strain-specific primers. Numbers at the top indicate the primer pairs. L, 100 bp DNA ladder; Lane 1, live LS06; Lane 2, heat-killed *L. sakei* LS06; Lane 3, KACC17865; Lane 4, KACC17868; Lane 5, KACC17871; Lane 6, KACC18352; Lane 7, KACC17864; Lane 8, KACC16119.

Three unique genetic markers of *L. sakei* K040706 (K040706..2017, K040706..2018, and K040706..2019) were subjected to PCR to check their specificities against live and heat-killed *L. sakei* K040706, and six other *L. sakei* sp. The PCR was carried out at 55–57 °C. (Figure 3). The expected lengths of the PCR products of the genetic markers K040706..2017, K040706..2018, and K040706..2019 were 158, 160, and 170 bp, respectively. Amplicons appeared only in the live and heat-killed *L. sakei* K040706 samples, but not in the other samples. These results suggest that the designed primers bind the target sequences with high specificity.

**Figure 4:**
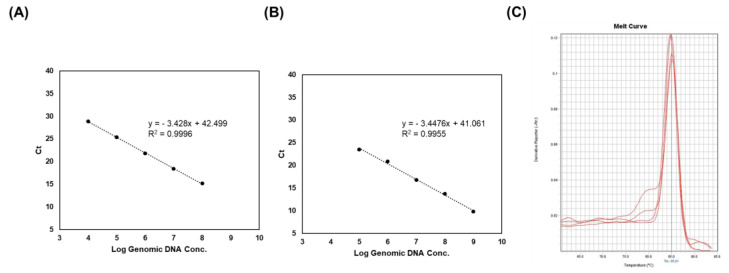
Real-time PCR analysis of genomic DNA, live, and heat-killed LS06 for a range of concentrations. (**A**) Standard curve of live *L. sakei*. (**B**) Standard curve of heat-killed LS06. (**C**) Melt curve generated by strain-specific primers in real-time PCR.

To evaluate the efficiency and limit of detection of PCR, a RT-PCR assay was conducted using the 2018 primer pair, since it showed a high density of PCR bands (Figure 4). Strain-specific primers exhibited a linear plot with a range of 2.1 × 10^4^ to 10^8^ CFU/mL in the live sample (Figure 4A). In addition, DNA samples from the heat-killed LS06 showed a limit of detection of 2.1 × 10^5^ CFU/mL (in triplicate) (Figure 4B). All R^2^ values (correlation coefficients) were >0.99 and the efficacies of the PCR were >0.9. Melt curve analysis indicated only one melting peak varying from 85 to 86 °C (Figure 4C). These results implied that the PCR product was generated by specific primer binding.

### 3.3. Amelioration of Thymus Index Alteration in CTX-Treated Mice by Heat-Killed LS06 Administration

**Figure 5:**
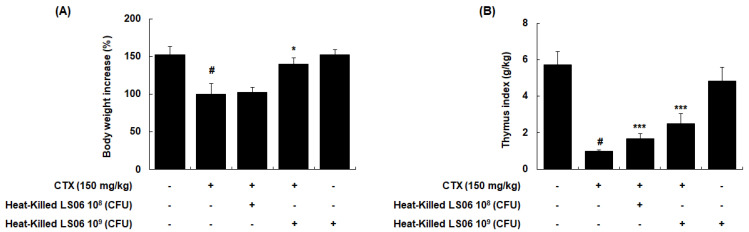
Comparison of (**A**) body weight and (**B**) thymus index between CTX and heat-killed LS06 treatment in mice. Thymus index was calculated as thymus weight (g)/body weight (kg). Data are presented as the mean ± SEM (*n* = 10). # *p* < 0.05 vs. Con group; * *p* < 0.05, *** *p* < 0.001 vs. CTX group.

To determine the immune response improvement of heat-killed LS06 in CTX-treated mice, we evaluated whether oral administration of heat-killed LS06 (10^8^ or 10^9^ CFU/day) recovered the CTX-reduced body weight and thymus index in mice. CTX injection (150 mg/kg, i.p.) significantly reduced body weight, while a high-dose heat-killed LS06 (10^9^ CFU/day, p.o.) restored the bodyweight (*p* < 0.05, Figure 5A). In addition, high- and low-dose of heat-killed LS06 significantly improved the thymus index in CTX-treated mice (Figure 5B).

### 3.4. Heat-Killed LS06-Modulated Immune Cells in CTX-Treated Mice

**Figure 6:**
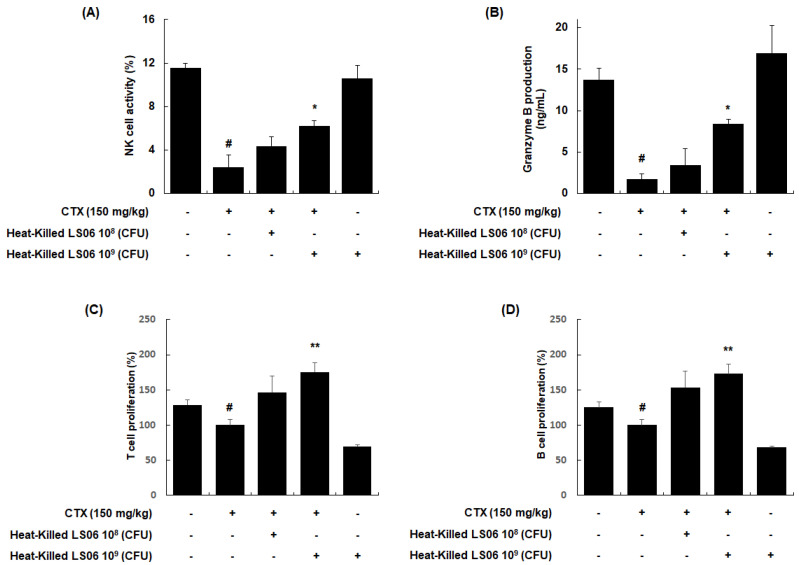
Effects of heat-killed LS06 on the regulation of immune cells in CTX-treated mice. Heat-killed LS06 (10^8^ or 10^9^ CFU/day, p.o.) were provided for 20 days and the splenocytes were prepared from isolated spleen of sacrificed mice. (**A**) NK cell cytotoxic activity and (**B**) granzyme B production (**C**,**D**) T and B cell proliferation. Data are presented as the mean ± SEM (*n* = 10). # *p* < 0.05 vs. Con group; * *p* < 0.05, ** *p* < 0.01 vs. CTX group.

NK cells are cytotoxic and secrete granzyme B to lyse the target cancerous and virus-infected cells [18]. We investigated whether the heat-killed LS06 modulates the NK cell cytotoxicity against YAC-1 target cells. Our data showed that NK cell activity in the splenocytes was significantly reduced in CTX-treated mice compared to the vehicle-treated control group (2.40 ± 1.11% vs. 11.54 ± 0.45%, respectively, *p* < 0.001, Figure 6A). However, the NK cell activity in splenocytes was significantly enhanced upon high-dose heat-killed LS06 (10^9^ CFU/day, p.o.) treatment of CTX-treated mice compared to only CTX-treatment in mice (6.23 ± 0.47% vs. 2.40 ± 1.11%, respectively, *p* < 0.05). Similarly, high-dose heat-killed LS06 (10^9^ CFU/day, p.o.) treatment of CTX-treated mice significantly elevated granzyme B production in splenocytes than in only CTX-treated mice (8.42 ± 0.58% vs. 1.71 ± 0.66%, respectively, *p* < 0.05, Figure 6B). We further evaluated the effects of heat-killed LS06 on Con A- or LPS-induced splenocyte proliferation in CTX-immunosuppressed mice [19]. As expected, the CTX group significantly suppressed the proliferative responses of T and B cells compared to the Con group (Figure 6C,D). Meanwhile, the administration of heat-killed LS06 at 10^9^ CFU/day significantly elevated the Con-A- or LPS-induced T cell proliferation by up to 176% or B cell proliferation by up to 173%, respectively, over the only CTX-treated group.

### 3.5. Heat-Killed LS06-Regulated Th1 Cytokines in Splenocytes of CTX-Treated Mice

**Figure 7:**
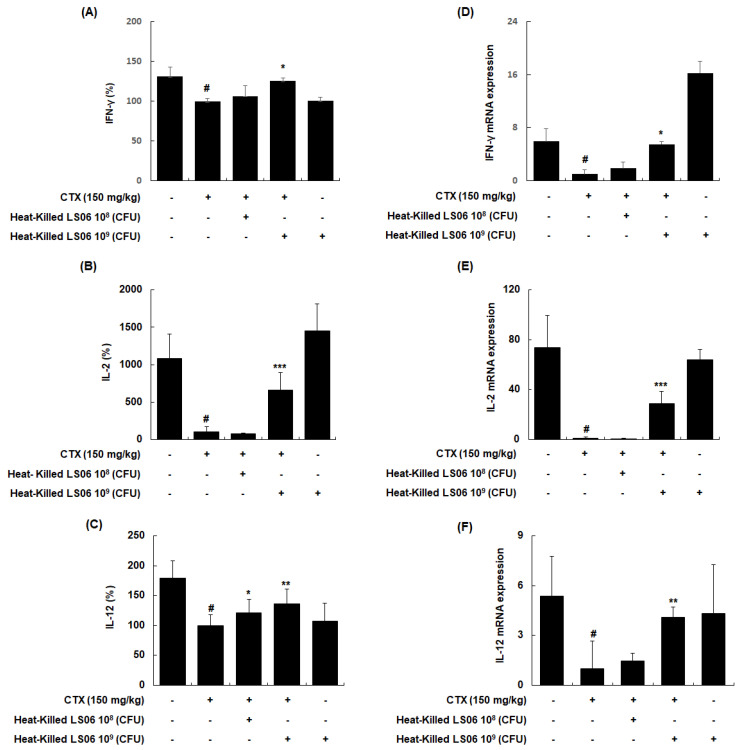
Effects of heat-killed LS06 on the expression level of Th1-related cytokines production and mRNA in splenocytes isolated from CTX-treated mice. (**A**–**C**) The production and (**D**–**F**) mRNA expression levels of the Th1-related cytokines. Data are presented as mean ± SEM (*n* = 10). # *p* < 0.05 vs. Con group; * *p* < 0.05, ** *p* < 0.01, and *** *p* < 0.001 vs. CTX group.

To explore whether heat-killed LS06 control T helper (Th) cells, the effects of heat-killed LS06 on Th1-related cytokines in splenocytes were determined. CTX treatment significantly reduced the production and mRNA expression of IFN-γ, IL-2, and IL-12, whereas the administration of heat-killed LS06 significantly increased the protein and mRNA expression levels of Th1 cell-related cytokines in splenocytes isolated from CTX-immunosuppressed mice (Figure 7). Interestingly, heat-killed LS06 provision did not affect IL-10, IL-13, and IL-4 productions, indicating the Th2-related cytokines (data not shown). Our data show that the Th1-activating effect of heat-killed LS06 is mediated by increasing cytokine production and mRNA expression in CTX-treated mice.

### 3.6. Effects of Heat-Killed LS06 on Microbiota Composition in CTX-Treated Mice

**Figure 8:**
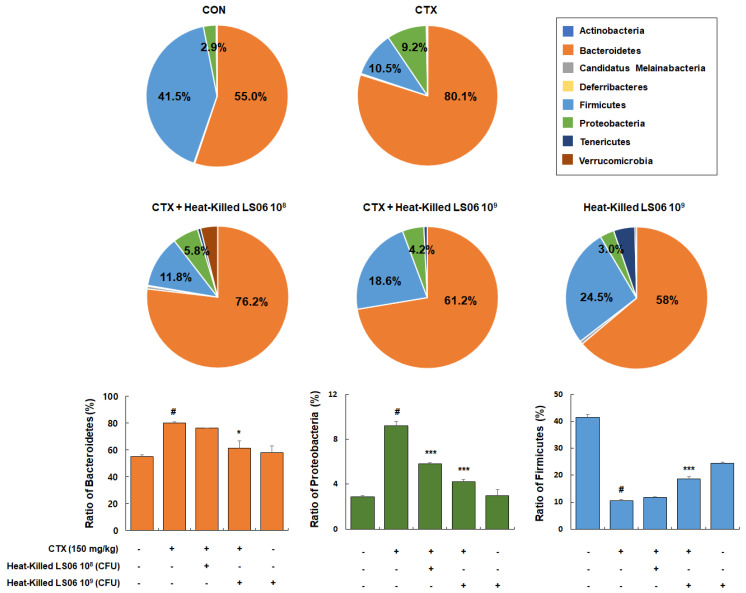
Effects of heat-killed LS06 treatment on the composition of microbiota in CTX-treated mice. Genomic DNA was analyzed for bacterial composition using 16S rRNA gene sequencing. Data are presented as mean ± SEM (*n* = 10). # *p* < 0.05 vs. Con group; * *p* < 0.05 and *** *p* < 0.001 vs. CTX group.

Besides being a potent immunosuppressant, CTX also reduces the intestinal tight and adherens junctions and triggers the increase in pathogenic bacteria. Hence, it is important in modulating the intestinal microbiota composition and immune function [18]. We thus determined the effects that heat-killed LS06 provide on the gut microbiota composition in CTX-treated mice. Dominant bacteria phyla mainly consist of Bacteroidetes (55.0% ± 1.3%), Firmicutes (41.5% ± 1.1%), and Proteobacteria (2.9% ± 0.1%, Figure 8). The CTX-treated group showed relative ratio of Bacteroidetes (80.1% ± 1.0%), Firmicutes (10.5% ± 0.5%), and Proteobacteria (9.2% ± 0.4%), while high-dose heat-killed LS06 supplementation to CTX-treated mice significantly altered relative abundance ratio of Bacteroidetes (61.2% ± 5.6%), Proteobacteria (4.2% ± 0.2%), and Firmicutes (18.6% ± 0.8%).

**Figure 9:**
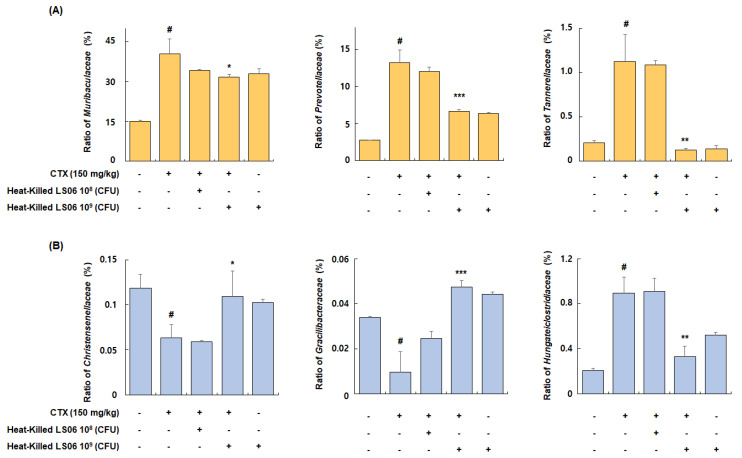
Effects of heat-killed LS06 treatment on family’s composition in CTX-treated mice. Relative abundance ratio of families of (**A**) Bacteroidetes and (**B**) Firmicutes. Data are presented as mean ± SEM (*n* = 10). # *p* < 0.05 vs. Con group; * *p* < 0.05, ** *p* < 0.01, and *** *p* < 0.001 vs. CTX group.

Among families of the dominant Bacteroidetes phylum, the relative abundance ratio of *Muribaculaceae*, *Prevotellaceae*, and *Tannerellaceae* increased in the CTX-treated group compared to the Con group (40.4 ± 5.6%, 13.2 ± 1.7%, and 1.1 ± 0.3%, respectively), but these increases were significantly suppressed by the administration of high-dose heat-killed LS06 (31.8 ± 0.9%, 6.6 ± 0.2%, and 0.1 ± 0.02%, respectively) (Figure 9A). Furthermore, among bacterial families of the Firmicutes phylum, the relative abundance ratio of *Christensenellaceae* and *Gracilibacteraceae* significantly suppressed in the CTX-treated group compared to the Con group (0.06 ± 0.01% and 0.01 ± 0.009%, respectively), but this suppression was improved by high-dose heat-killed LS06 supplementation (0.11 ± 0.03% and 0.05 ± 0.003%, respectively) (Figure 9B). Conversely, the relative abundance rate of *Hungateiclostridiaceae*, which was increased by CTX treatment (0.89 ± 0.14%), was significantly reduced following a high-dose heat-killed LS06 administration (0.33 ± 0.09%). These results suggest that heat-killed LS06 administration could effectively improve the CTX-modulated gut microbiota composition and mediate immunomodulation.

## 4. Discussion

Probiotics are microorganisms that generate a wide range of beneficial biological responses in their host, including immunostimulatory effects [20]. In our previous study, live K040706 regulated immune and inflammatory responses in vitro and in vivo experiments [11,12]. Although probiotics are primarily live microbial agents, several concerns remain about the possibility of unwanted side effects, including a higher risk of infection in sick or immunocompromised patients and very young individuals, ignition of an inflammatory response, and translocation to the locally draining tissues and blood [21]. Another concern is the safety aspects of bacteria including the acquisition of virulence genes or antimicrobial resistance [22]. These problems lead to develop alternative agents, such as probiotics killed by heat. Previous studies have demonstrated that oral rehydration therapy by *Latilactobacillus* *acidophilus* LB is effective in the treatment of children with acute diarrhea [23] and LPS-induced inflammation can be modulated by heat-killed *Latilactobacillus* *rhamnosus* GG [24]. Other studies have shown that heat-killed probiotics exhibit immunomodulatory properties under various temperature and time conditions [25,26].

There are several methods to identify LAB in probiotics and dairy products. However, these methods have only been used on living bacteria. In this study, we present a method of detecting heat-killed LAB by using novel genetic markers of *L. sakei* K040706 and strain-specific PCR primers based on comparative genomics. Genomic DNA can be extracted after heat inactivation and detected by real-time PCR.

Before the spread of whole-genome sequencing, random sequence (RAPD-PCR), amplification of repetitive sequences (Rep-PCR), or amplified length polymorphism analysis were used to classify bacteria. These methods are time-consuming, labor-intensive, and have low reproducibility [27]. Hence, to screen strain-specific sequences, pairwise alignment of the genome and pan-genome analysis is a more powerful tool [28,29,30]. In this study, the unique genetic markers (K040706..2017, K040706..2018, and K040706..2019) of *L. sakei* K040706 were selected and encoded into DNA modification methylase and two hypothetical proteins, respectively. Using BLASTN search, these proteins were identified as coding sequences with low DNA sequence homology. When the strain-specific primer was subjected to PCR with other *L. sakei* strains, K040706 was successfully validated.

The thymus and spleen are two vital organs in human immune regulation. The thymus is an important regulator of peripheral immune organs and immune cells, providing a place for T cell development [31]. T cells are involved in immune responses against pathogens and some cancers. In contrast, the spleen is the production site of T and B cells and can synthesize antibodies against blood-borne antigens [32]. It is well known that CTX-induced immunosuppression leads to weight loss in the thymus and spleen, reflecting the impairment of immune cells and suppressing the number of T and B cells [33,34]. In the present study, oral administration of heat-killed LS06 improved the CTX- reduced body weight, thymus index, and splenic T and B cell proliferation, indicating the recovery of immune function. Stimulating immune cells secrete cytokines, which are small-molecule soluble proteins with immune functions. After T cell differentiation into effector cells, CD4^+^ Th cells are divided into distinct cell types, Th1 and Th2, which secrete different types of cytokines to modulate the immune system. Th1 cells produce pro-inflammatory cytokines such as IFN-γ, IL-2, and IL-12, which stimulate phagocytosis, destroy pathogens, and are involved in organ-specific autoimmune diseases [35]. In contrast, Th2 cytokines produce the cytokines IL-4, IL-10, and IL-13, which stimulate the production of antibodies directed toward large extracellular parasites, including allergies. Hence, the balance between Th1 and Th2 cytokine production can determine the outcome of an immune response. CTX administration leads to intestinal cellular immune dysfunction, resulting in Th1/Th2 imbalance. In this study, we demonstrated that although heat-killed LS06 supplementation had no significant effect on inducing Th2-related cytokines production, the mRNA and protein expression of Th1-related cytokines recovered. This indicates that Th1 cytokines are more potently affected by heat-killed LS06 than Th2. We thus considered that heat-killed LS06-related immune improvement could modulate the Th1/Th2 balance in CTX-induced immunosuppressed mice.

The gut microbial ecosystem harbors trillions of taxonomically-diverse microorganisms that perform a range of physiological functions in the gastrointestinal tract of the host [36]. Imbalances in the gut microbial community are associated with various clinical conditions, including CTX-induced immunosuppression. CTX is a medicine for chemotherapy with evidence of efficacy and safety. However, a high-dose of CTX can lead to hepatotoxicity, mucosal barrier disruption, and an increase of pathogenic bacteria in the intestine [37]. In the present study, CTX changed the microbiota composition, including alteration in the numbers of Bacteroidetes and Firmicutes, which are the two most predominant phyla and account for almost 90% of total bacteria [37]. The CTX-induced changes in Bacteroidetes and Firmicutes were consistent with previous studies suggesting that alteration of phylum abundance may promote immunosuppression and disruption of gut homeostasis [38]. However, the heat-killed LS06 reverted the composition of phyla in the immunosuppressed mice to normal. We sequentially analyzed the abundance of *Muribaculaceae*, *Prevotellaceae*, *Tannerellaceae*, *Christensenellaceae*, *Gracilibacteraceae*, and *Hungateiclostridiaceae*. Youlong et al. demonstrated that *Muribaculaceae* was positively related to immune traits, whereas *Prevotellaceae* and *Tannerellaceae* were negatively correlated with immune traits [38]. In addition, members of the *Tannerellaceae* family are most abundant in certain gastrointestinal disorders, such as Crohn’s disease [39]. Our study showed that CTX injection significantly increased the relative abundance of *Muribaculaceae*, *Prevotellaceae*, and *Tannerellaceae*, but heat-killed LS06 intervention reversed these changes. CTX administration has been reported to decrease the abundance of *Christensenellaceae*, one of five taxa regarded as a sign of a healthy gut [40,41]. However, the abundance of *Gracilibacteraceae* and *Hungateiclostridiaceae* in the gut microbiota after CTX injection has not been elucidated. We found that CTX administration also reduced the abundance of *Gracilibacteraceae* and *Hungateiclostridiaceae.* Further, *Christensenellaceae, Gracilibacteraceae,* and *Hungateiclostridiaceae* strains in CTX-treated mice significantly increased after oral administration of heat-killed LS06. Since the compositional diversity and abundance of microbiota are fundamental to the maintenance of the immune system, further studies are needed to determine how the heat-killed LS06 performs its immunostimulatory activity to maintain immune homeostasis.

## 5. Conclusions

In conclusion, we demonstrated the development of a strain-specific primer and the successful quantification of heat-killed LS06 with high specificity. Further, we showed that the oral administration of heat-killed LS06 ameliorated CTX-induced suppression of immunity by recovering body weight, thymus index, immune cell proliferation, and Th1-related cytokines productions. Heat-killed LS06 improves immune responses which may be related to an alteration of the microbiota composition to normal. Therefore, heat-killed *L. sakei* K040706 may be utilized as an ingredient of functional food for health benefits via immune modulation and regulation of gut microbiota.

## Figures and Tables

**Table 1:** *Lactilactobacillus sakei* strains used in this study.

Strain Number	Source
K040706	Lab stock
KACC17865	KACC (Korean Agricultural Culture Collection)
KACC17868	
KACC17871	
KACC18352	
KACC17864	
KACC16119	

**Table 2:** The list of primer sequences for strain-specific qRT-PCR.

Primer	Sequence (5′ to 3′)	Position *	Amplicon Size (bp)
2017_F	AAGAGTTCGGATGGCAGCAA	1,976,642–1,976,623	158
2017_R	CGCTATCCGATAAGCTCGCA	1,976,485–1,976,504	
2018_F	ATGGGTAAAATGATTCACTCGAAATATG	1,976,869–1,976,842	160
2018_R	TTATCTATTGGCCACTCTTCTATT	1,976,711–1,976,734	
2019_F	GAAAAGGGATGCGATTGCCG	1,977,050–1,977,031	170
2018_R	ATCACCCACCACTTGCCAAT	1,976,875–1,976,894	

* The positions are enumerated based on the complete genome sequence of *L. sakei* under accession number Sequence Read Archive (SRA): https://www.ncbi.nlm.nih.gov/sra/PRJNA758190, accessed on 26 August 2021.

## Data Availability

Not applicable.

## Supplementary Materials

The following are available online at https://www.mdpi.com/article/10.3390/foods10123034/s1, Table S1: The list of primer sequences for qRT-PCR.
