# Supplementary material for: Strain-Specific Identification and In Vivo Immunomodulatory Activity of Heat-Killed Latilactobacillus sakei K040706
Source: Foods. 2021 Dec 7;10(12):3034. doi: 10.3390/foods10123034 (PMC8701173; doi:10.3390/foods10123034)
Supplement: Supplementary file 1 [file foods-10-03034-s001.zip › foods-1480033-supplementary.pdf]

Table S1. The list of primer sequences for qRT-PCR.

Gene	Forward	Reverse	Tm (°C)
IFN-γ	GCTTCCTGAGGCTGGATTC	TACCTTCTTCAGCAACAGCAAG	55
IL-2	TCCTGGGGAGTTTCAGGTTC	CTCTACAGCGGAAGCACAGC	55
IL-12	TCTGCAGAGAAGGTCACACT	ATGAAGAAGCTGGTGCTGTA	55
β-actin	ATCACTATTGGCAACGAGCG	TCAGCAATGCCTGGGTACAT	55

The oligonucleotide primers were designed using Primer3, and the specificity checking module used BLAST.
